# SDF-1α/MicroRNA-134 Axis Regulates Nonfunctioning Pituitary Neuroendocrine Tumor Growth *via* Targeting VEGFA

**DOI:** 10.3389/fendo.2020.566761

**Published:** 2020-12-09

**Authors:** Xiaoyu Wang, Yuanjian Fang, Yunxiang Zhou, Xiaoming Guo, Ke Xu, Chenguang Li, Jianmin Zhang, Yuan Hong

**Affiliations:** ^1^ Department of Neurosurgery, The Second Affiliated Hospital, School of Medicine, Zhejiang University, Hangzhou, Zhejiang, China; ^2^ Department of Surgical Oncology, The Second Affiliated Hospital, School of Medicine, Zhejiang University, Hangzhou, Zhejiang, China; ^3^ Brain Research Institute, Zhejiang University, Hangzhou, Zhejiang, China; ^4^ Collaborative Innovation Center for Brain Science, Zhejiang University, Hangzhou, Zhejiang, China

**Keywords:** SDF-1α (CXCL12), vascular endothelial growth factor A (VEGFA), proliferation, invasion, microRNA-134(miR-134), nonfunctioning pituitary neuroendocrine tumor (NF-PitNET)

## Abstract

**Background:**

Nonfunctioning pituitary neuroendocrine tumor (NF-PitNET) is difficult to resect. Except for surgery, there is no effective treatment for NF-PitNET. MicroRNA-134 (miR-134) has been reported to inhibit proliferation and invasion ability of tumor cells. Herein, the mechanism underlying the effect of miR-134 on alleviating NF-PitNET tumor cells growth is explored.

**Methods:**

Mouse pituitary αT3-1 cells were transfected with miR-134 mimics and inhibitor, followed by treatment with stromal cell-derived factor-1α (SDF-1α) *in vitro*. MiR-134 expression level: we used quantitative real-time PCR (qRT-PCR) to detect the expression of miR-134. Cell behavior level: cell viability and invasion ability were assessed using a cell counting kit-8 (CCK8) assay and Transwell invasion assay respectively. Cytomolecular level: tumor cell proliferation was evaluated by Ki-67 staining; propidium iodide (PI) staining analyzed the effect of miR-134 on cell cycle arrest; western blot analysis and immunofluorescence staining evaluated tumor migration and invasive ability. Additionally, we collected 27 NF-PitNET tumor specimens and related clinical data. The specimens were subjected to qRT-PCR to obtain the relative miR-134 expression level of each specimen; linear regression analysis was used to analyze the miR-134 expression level in tumor specimens and the age of the NF-PitNET population, gender, tumor invasion, prognosis, and other indicators.

**Results:**

*In vitro* experiment, miR-134 was observed to significantly inhibit αT3-1 cells proliferation characterized by inhibited cell viability and expressions of vascular endothelial growth factor A (VEGFA) and cell cycle transition from G1 to S phase (*P* < 0.01). VEGFA was verified as a target of miR-134. Additionally, miR-134-induced inhibition of αT3-1 cell proliferation and invasion was attenuated by SDF-1α and VEGFA overexpression (*P* < 0.01). In primary NF-PitNET tumor analysis, miR-134 expression level was negatively correlated with tumor invasion (*P = 0.003*).

**Conclusion:**

The regulation of the SDF-1α/miR-134/VEGFA axis represents a novel mechanism in the pathogenesis of NF-PitNETs and may serve as a potential therapeutic target for the treatment of NF-PitNETs.

## Introduction

Pituitary tumors are common and account for 10–15% of primary intracranial neoplasms, and 30% originate from gonadotroph (1). Nonfunctioning pituitary adenomas are usually of gonadotroph origin (2) and cannot secrete biologically active hormones (3); however, silent corticotroph, poorly differentiated PIT1-lineage, true null cell tumors, or other silent tumors have also been described, and they often have a more aggressive behavior (4). Recently, a new terminology, pituitary neuroendocrine tumor (PitNET), which is proposed by the International Pituitary Pathology Club, proposed to replace adenoma to better reflect the similarities between adenohypophyseal and neuroendocrine tumors of other organs (5). Generally, patients with NF-PitNET often present with hypopituitarism and visual field defects, due to the compression effect of development of large adenomas (6, 7). Compared with small pituitary tumors, large and invasive pituitary tumors are more difficult to resect, and may be companied with high risks of complications, such as hypothalamic injury, visual impairment, vascular injury, cerebrospinal fluid leakage, pituitary dysfunction, infection, and reoperation or radiation therapy due to recurrence. Currently, surgical resection remains the major treatment for NF-PitNET (8). No effective drug is available for the treatment of NF-PitNET, which seriously affects the outcome of NF-PitNET patients. Therefore, it is urgent to further explore the underlying mechanisms of pituitary tumorigenesis, and provide a theoretical basis for finding new therapeutic targets for NF-PitNET.

MicroRNAs (miRNAs) are a class of 20–25 base-pair (bp)- long noncoding RNAs that regulate gene expression by severing the mRNA to target gene or inhibiting translation of target genes (9). Cheunsuchon et al. (10) have confirmed that the specific expression of six microRNAs (miR-134, miR-299-5p, miR-329, miR-370, miR-377-5p, and miR-432) downstream of delta-like homologue 1–maternally-expressed gene 3 (DLK1-MEG3) gene cluster were lost or reduced in NF-PitNET, but expressed in normal pituitary and other types of pituitary tumors. This finding suggests that the loss or reduction of expression of these six types of miRNAs may play a key role in the occurrence of NF-PitNET.

Previous studies have shown that overexpression and interaction of stromal cell derived factor-1α (SDF-1α) and its receptor CXCR4 play an important role in pituitary cell proliferation and tumorigenesis (11). SDF-1α secreted by fibroblasts plays a key role in the enhancement of NF-PitNET cell proliferation, invasion, migration, transformation, and tumor formation (12). The molecular mechanism of action of SDF-1α in NF-PitNET is still not fully understood. We hypothesize that SDF-1α may play a role in promoting tumorigenesis by affecting the expression of miRNAs in NF-PitNET.

By stimulating the αT3-1 cells with exogenous recombinant SDF1α, we previously found that the expression level of miR-134 in αT3-1 cells was significantly decreased by qRT-PCR (13). MiR-134 can regulate the proliferative activity of pituitary folliculostellate cells and lung cancer tumor cell lines, and arrest cell cycle (14, 15). VEGFA is one of the downstream target proteins of miR-134 (13). It can chemoattract endothelial progenitor cells in tumor stroma or other sites, and stimulate the survival of neovascularization in tumor tissues, thereby promoting tumor cell proliferation and distant migration. Also, clinical studies have shown a significant negative correlation between VEGFA expression and tumor prognosis (16). Shan et al. (17) found pituitary tumor cells increased HIF-1α expressing under hypoxic conditions, and stimulate VEGFA generation, thus promoting the development of pituitary tumor. Meanwhile, Samuel et al. (18) found that SDF-1α/CXCR4 can activate VEGFA to mediate the formation of new blood vessels in tumor tissues through HIF-1α, thereby promoting tumor growth in human colorectal cancer cells.

Therefore, based on results of previous studies and our preliminary experiment, we hypothesized that in NF-PitNET, SDF-1α may regulate the production of its target protein VEGFA by regulating the level of miR-134, and subsequently mediate tumor cell proliferation, migration, and invasion. In this study, because of the high conservation of miR-134 between human and mouse, mouse pituitary αT3-1 cells were used to determine the effect of SDF-1α on miR-134 expression *in vitro* model, and detect the regulation of miR-134 on VEGFA synthesis, proliferation, migration, and invasion in tumor cells treated with SDF-1α. In addition, by collecting human NF-PitNET specimens, the relationship between miR-134 expression level and tumor invasiveness and prognosis were analyzed to reveal the mechanism of NF-PitNET development under the action of SDF-1α which may serve as a potential therapeutic target for the treatment of NF-PitNET patients.

## Methods

### Human Tissue Samples

With approval of Ethics Committee, and written informed consent by patients or their guardians, a total of 29 human NF-PitNETs specimens were obtained from the Department of Neurosurgery, the Second Affiliated Hospital, School of Medicine, Zhejiang University from December 2015 to January 2019. All specimens were obtained from patients who underwent tumor resection or biopsy in our hospital, and Pituitary tumors are diagnosed as “nonfunctioning” in the absence of clinical or biochemical evidence of tumor-related hormone excess. One part of the tissue fragments was frozen in liquid nitrogen and stored at −80°C for qRT-PCR. Another part was formalin‐fixed, paraffin‐embedded for pathological analysis. The clinical features of patients including age, sex, expression of Ki-67, and invasion. Radiological signs and/or surgical detection of cavernous or sphenoid sinus invasion, or diaphragm or floor of sellar invasion should be highly suspected of invasive NF-PitNET. Resistance to medical treatment and multiple recurrences despite standard therapies were defined to invasive NF-PitNET (19). Ki-67 ≥3% was considered high proliferation. All patients were first time for the surgery and no patients received medical therapy before the specimens’ collection.

### Cell Culture

The mouse gonadotroph αT3-1 cell line was cultured in Dulbecco’s modified Eagle medium (DMEM) (Gibco, Shanghai, China) supplemented with 10% fetal bovine serum (FBS) with 100 U/ml penicillin and 100 μg/ml streptomycin (Invitrogen, Shanghai, China). All cell lines were incubated at 37° in a standard mixture of 95% air and 5% CO2. The cells were observed as adherent cells under the microscope, and the live cell rate of trypan blue staining was over 95%.

### Quantitative Real-Time PCR Analysis

Total RNA was extracted from tissue samples cells by using TRIzol reagent (Invitrogen, USA). Then cDNA synthesis from the isolated total 1 µg RNA was performed using NCode VILO miRNA cDNA synthesis Kit (Invitrogen, USA). Primer sequences and PCR conditions were described below: hsa-miR-134a: forward, 5′-TGTGACTGGTTGACCAGAGGGG-3′; reverse primers are provided by NCode EXPRESS SYBR GreenER miRNA qRT-PCR kit (Invitrogen, A11193-052, Shanghai, China). mmu-miR-134: forward, 5’ ACACTCCAGCTGGGTGTGACTGGTTGACCA3’; reverse, 5’CTCAACTGGTGTCGTGGAGTCGGCAATTCAGTTGAGCCCCTC3’; U6: forward, CTCGCTTCGGCAGCACA; reverse, FCTCGCTTCGGCAGCACA. Quantitative real-time PCR was performed with NCode EXPRESS SYBR GreenER miRNA qRT-PCR Kit (Invitrogen, Shanghai, China), according to the manufacturer’s instructions, on the ABI Prism 7300 SDS system (ABI, USA). Small nuclear RNA U6 was used as the endogenous control. The expression levels of miRNAs were determined using the 2-ΔΔCt method (20). All samples were analyzed in triplicate. The data was analyzed by the ABI Prism 7300 SDS Software.

### Plasmid Construction, Transfection, and SDF-1α Treatment

mmu-miR-134–5p mimic, mmu-miR-134–5p inhibitor and blank vector control were synthesized by GenePharma (Shanghai, China). These constructs were transfected into cells by using Lipofectamine 2000 according to manufacturer instructions (Thermo Scientific, Waltham, USA). Exogenous recombinant SDF-1α cytokine (PeproTech Ltd. China) was added to the SDF-1α stimulation group at a final concentration of 20 ng/ml, and an equal volume of phosphate buffer saline (PBS) containing 0.1% BSA was added to the control group (for preparation of SDF-1α solution). Mouse cell lines were grouped into: mmu-miR-134–5p inhibitor + SDF-1α, mmu-miR-134–5p inhibitor + PBS, mmu-miR-134–5p mimic + SDF-1α, mmu-miR-134–5p mimic + PBS, blank vector control + SDF-1α, and blank vector control + PBS.

### Cell Cycle and Cell Proliferation Assay

After treatment, cells were collected and incubated with RNase A (50 µg/ml) and propidium iodide (PI) (50 µg/ml) (MultiSciences, China). Flow detection was completed within 24 h after dyeing was completed. The fluorescence was detected by a FACSCalibur flow cytometer (BD, Shanghai, China) and subsequent cell cycle analysis using FLOWJO software (Treestar, USA). Cell proliferation analysis was performed with the Cell Counting Kit-8 (CCK8) (Beyotime, Shanghai, China). A total 3 × 10^3^ cells cultured in each well of 96-well plates for 24, 48, and 72 h. Then, 10 μl of CCK8 was added to each well and cells were incubated at 37°C for 1 h. The absorbance was detected at 450 nm and measured using an automatic plate analyzer (Bio-Rad Lab, Hercules, CA, USA).

### Transwell Invasion Assay

Cell invasion assay was performed using Transwell plates (Corning, USA) with 8-µm-pore size membranes with Matrigel used to measure the invasion ability of cells. NF-PitNET cells with appropriate transfection treatments were harvested after 48 h and seeded with serum-free medium (5 × 10^5^ cells in 300 μl) into the upper chamber. Bottom wells were filled with complete medium. After 24 h, the cells on the upper surface of the membrane were removed by cotton swabs. The cells that moved through the membrane were fixed with 4% paraformaldehyde and stained with 1% crystal violet. Images of the invasion and migration cells were photographed under a microscope. All of the experiments were performed in triplicate.

### Western Blot Analysis

All cells were lysed in RIPA-Buffer (Beyotime Biotechnology, Shanghai, China) supplemented with protease and phosphatase inhibitors (Thermo Scientific, USA) on ice for 30 min, followed by centrifugation at 12,000 g for 15 min. Protein concentrations were calculated using the Pierce BCA protein assay kit (Thermo Scientific, USA). Equivalent amounts of protein samples (25 µg/lane) were separated on 10% sodium dodecyl sulfate-polyacrylamide gel electrophoresis (SDS-PAGE) and subsequently transferred to polyvinylidene fluoride (PVDF) membranes. Membranes were blocked with 5% nonfat milk in Tris-buffered saline and 0.1% Tween 20 buffer and then incubated with primary antibody overnight at 4°C. Primary antibody including anti-VEGFA (Proteintech, MA5-13182), anti-ABCC1 (Abcam, ab91451), anti-MMP2 (Abcam, ab97779), anti-MMP9 (Abcam, ab38898), anti-Vimentin (Abcam, ab28151), anti-SNAIL (Cell Signaling Technology, #3879), GAPDH (Cell Signaling Technology, #5174), β-actin (ab179467). Membranes were incubated with goat anti-rabbit or anti-mouse HRP-conjugated secondary antibody (Beyotime Biotechnology, Shanghai, China) for 1 h at room temperature. Signal detection was performed using the ultrasensitive ECL plus Detection Reagent (Millipore, USA) and a ChemiDoc Touch Imaging System. The intensity of the bands was analyzed using the Quantity One software (Bio‐Rad, Hercules, CA, USA).

### Immunohistochemistry and Immunocytochemistry

The tissues were routinely fixed in 10% formalin for 8 to 24 h and embedded in paraffin. Five microns serial sections were deparaffinized in xylene and dehydrated in graded ethanol. Endogenous peroxidase activity was blocked with 0.3% hydrogen peroxide in methanol for 30 min. The tissues were incubated with anti-SDF-1α primary antibodies (1:100; Abcam) and anti-VEGFA primary antibodies (1:100; Abcam) at 4°C overnight. Brain tissue from epileptic patients were stained with anti-SDF-1α and anti-VEGFA were used for the negative control. The images were captured under a SP5/Leica confocal microscope with LAS AF Lite software. The cells grown on glass coverslips were fixed with 4% formaldehyde with for 10 min, and washed with PBS. Cells were permeabilized with PBS containing Triton X-100 (0.1–0.25%) for 10 min, and blocked with 1% BSA for 30 min. Then, cells were incubated with rabbit diluted primary antibody overnight at 4°C. After three washes in PBS, cells were incubated for 1 h with secondary antibody and incubated with hematoxylin and diaminobenzidine (DAB). Images were captured with an inverted microscope.

### Statistical Analysis

All the experiments were performed at least in technical triplicate and in three independent biological repeats. Results are represented as the mean ± SD. Two tailed Student’s t-test or Pearson’s analysis (for comparing data from two groups) or one-way analysis of variance (ANOVA) with Tukey’s *post hoc* test (for comparing data from multiple groups) was used to calculate statistical significance. All values were obtained using GraphPad Prism 5.0 and SPSS 17.0 software. Data are expressed as means ± SD. A value of P < 0.05 was considered to be statistically significant.

## Results

### Participant Characteristics

**Figure 1:**
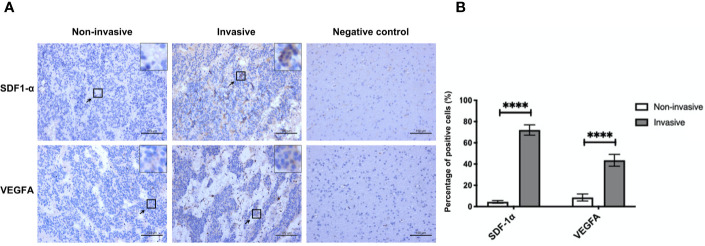
The expression of SDF-1α and VEGFA in invasive and non-invasive NF-PitNET. **(A)** Immunohistochemical staining assay of SDF-1α and VEGFA in invasive NF-PitNET and non-invasive NF-PitNET. Brain tissues from epileptic were used for the negative control. **(B)** The quantification of SDF-1α and VEGFA expression are shown between invasive and non-invasive NF-PitNET. Data were represented as means ± SD (n = 6). ^****^p < 0.0001 *vs.* non-invasive NF-PitNET group.

Of the 29 NF-PitNET patients, 19 (65.5%) were male and 10 (34.5%) were female. Age was 41.6 ± 3.6 years. miR-134 relative expression value was 5.0 ± 0.7. Twenty (69.0%) patients showed low Ki-67 expression with 9 (31.0%) patients showing high Ki-67 expression. Based on the criteria by Raverot et al. (19), “invasiveness” was defined resistance to medical treatment and multiple recurrences despite standard therapies. Seventeen (58.6%) NF-PitNETs were aggressive growth. All surgical specimens were clinically and pathologically confirmed as hormone-negative tumors. According to the median value of miR-134 (2.5), patients were divided into low miR-134 expression (n = 15, 51.7%) and high miR-134 expression groups (n = 14, 48.3%). The Ki-67 level and invasiveness were inversely correlated with the miR-134 expression (*P < 0.001* and *P = 0.003*, respectively, **Table 1**). In addition, we compared the expression of SDF-1α and VEGFA in invasive and non-invasive NF-PitNET tissue samples, and the results suggested that the expression levels of SDF-1α and VEGFA in invasive NF-PitNET are relatively high (*P < 0.0001*, **Figures 1A, B**).

**Table 1:** The correlation between clinical features of NF-PitNETs and the expression of miR-134.

Clinical features	Number (%)	Low miR-134 expression	
		Number (%)	*p* value
Age, years			
<42	13 (44.8)	7 (53.9)	NS
≥42	16 (55.2)	8 (50.0)	
Sex			
Male	19 (65.5)	10 (52.6)	NS
Female	10 (34.5)	5 (50.0)	
Ki-67 (%)			
<3	20 (69.0)	7 (35.0)	<0.001
≥3	9 (31.0)	8 (88.9)	
Aggressive			
Yes	17 (58.6)	11 (73.3)	0.003
No	12 (41.4)	4 (33.3)	

NS, No Significance.

### SDF-1α Treatment Attenuates the Expression of miR-134

**Figure 2:**
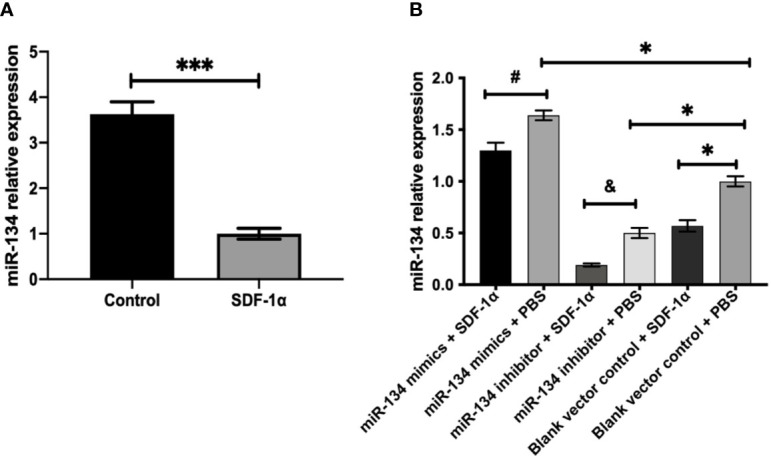
SDF-1α inhibits the expression of miR-134. αT3-1 cells were treated with SDF-1α, control, miR-134 mimics + SDF-1α, miR-134 mimics + PBS, miR-134 inhibitor + SDF-1α, miR-134 inhibitor + PBS, black vector control + SDF-1α, black vector control + PBS, respectively. **(A, B)** MiR-134/U6 relative level in αT3-1 cells by qRT-PCR. Data were represented as means ± SD (n = 3). ***p < 0.001 *vs.* control group. **^#^**p < 0.05 *vs.* miR-134 mimics + PBS group. ^&^ p < 0.05 *vs.* miR-134 inhibitor + PBS group. ^*^p < 0.05 *vs.* black vector control + PBS group.

Mouse pituitary αT3-1 cells were used to verify the effect of SDF-1α on the miR-134. QRT-PCR indicated that the expression level of miR-134 in the group treated with SDF-1α was significantly lower than that in the control group (*P < 0.001*, **Figure 2A**). Furthermore, the expression of miR-134 was similarly downregulated by SDF-1α in miR-134–5p inhibitor, miR-134–5p mimic, and blank vector control groups (*P < 0.05*, **Figure 2B**). The results indicated that SDF-1α was able to alleviate miR-134 expression.

### SDF-1α Promotes Cell Cycle Transition, Viability, and Proliferation *via* Suppressing the Expression of miR-134

**Figure 3:**
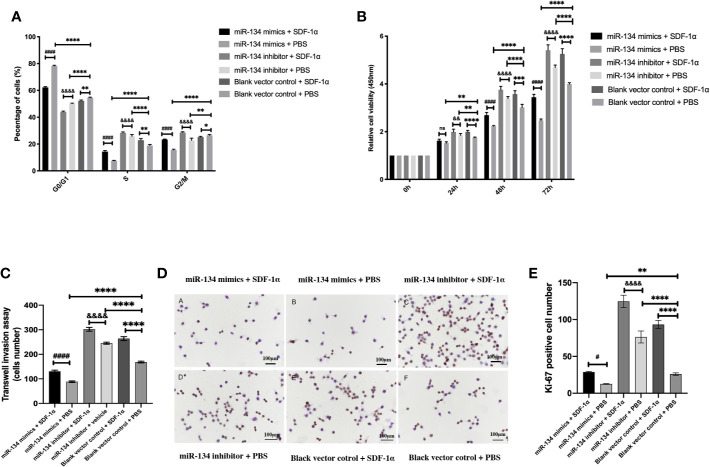
Effects of SDF-1α/miR-134 on cell cycle transition, proliferation, and viability. MiR-134 mimics or inhibitor was transiently transfected into αT3-1 cells. After 48 h transfection, αT3-1 cells were treated with 20 ng/ml SDF-1α, equal volume of PBS, and black vector control respectively. **(A)** Cell cycle distribution using flow cytometry. **(B)** Cell viability using CCK-8 assay. **(C)** Analysis of cell invasion using transwell assay. **(D)** Immunohistochemical staining assay of Ki-67. **(E)** Positive Ki-67 cell was counted manually by Image J. Data were represented as means ± SD (n = 3). **^####^**p < 0.0001 and **^#^**p < 0.05 *vs.* miR-134 mimics + PBS group. **^&&&&^**p < 0.0001 and **^&&^**p < 0.01 *vs.* miR-134 inhibitor + PBS group. ^****^p < 0.0001, ^**^p < 0.01 and ^*^p < 0.05 *vs.* black vector control + PBS group.

As presented by cell cycle analysis, αT3-1 cells treated with miR-134 mimics seemed to get trapped in G0/G1phase, and this effect was attenuated by SDF-1α treatment (*P* < 0.01). In contrast, cells treated with miR-134 inhibitor and SDF-1α had more cells in S phase, followed by cells treated with miR-134 inhibitor only (*P* < 0.01, **Figure 3A**). The results of CCK8 test to compare the proliferation ability of each group showed treatment with miR-134 inhibitor and SDF-1α increased the cell viability at 24, 48, and 72 h after treatment compared to other groups. Meanwhile, miR-134 mimics decreased the cell viability at 24, 48, and 72 h after treatment. Though the significance shown between all groups at 24, 48, and 72 h, it was found to be most remarkable at 72 h (*P* < 0.01, **Figure 3B**). Similar with the results of cell cycle analysis and CCK8 assay, treatment with SDF-1α and miR-134 inhibitor resulted in the most invasive cells, with SDF-1α or miR-134 inhibitor only following, which indicated that SDF-1α or miR-134 inhibitor increased the cell invasion ability (*P* < 0.01). While miR-134 mimics significantly decreased the cell invasion ability compared to other groups (*P* < 0.01, **Figure 3C**). Furthermore, the data of immunohistochemistry (Ki-67) confirmed that the miR-134 expression was negatively related with NF-PitNET cell proliferation, and SDF-1α can inhibit miR-134 expression which subsequently promoted proliferation (*P* < 0.05, **Figures 3D, E**).

### SDF-1α Boosts the Expression of VEGFA and Proliferation/Invasion-Related Downstream Protein of αT3-1 Cells *via* Repressing miR-134 Expression

**Figure 4:**
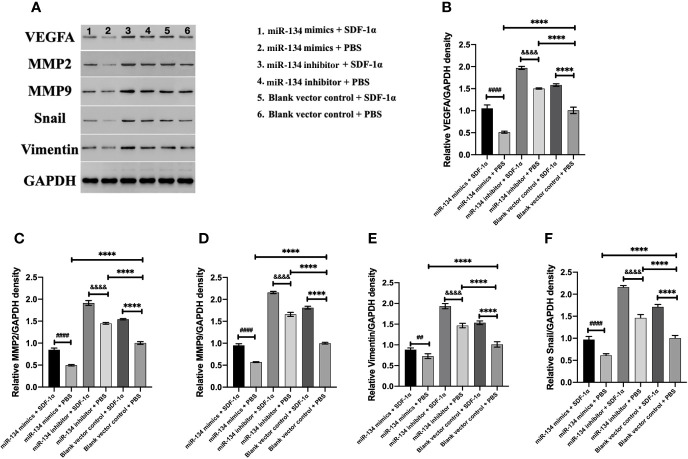
The expression of VEGFA and proliferation/invasion-related downstream protein of αT3-1 cells. **(A)** Schematic diagram of the target protein band. **(B)** The quantification of VEGFA expressions using western blot analysis. **(C)** The quantification of MMP2 expressions using western blot analysis. **(D)** The quantification of MMP9 expressions using western blot analysis. **(E)** The quantification of Vimentin expressions using western blot analysis. **(F)** The quantification of Snail expressions using western blot analysis. Data were represented as means ± SD (n = 5). ^####^p < 0.0001 and ^##^ p < 0.01 *vs.* miR-134 mimics + PBS group. ^&&&&^ p < 0.0001 *vs.* miR-134 inhibitor + PBS group. ^****^p < 0.0001 *vs.* black vector control + PBS group.

Previous study showed that MMP2/9, Snail, and Vimentin were considered as the proliferation invasion markers in pituitary growth (21–23). In the analysis of western blot result, we found miR-134 inhibitor + SDF-1α group has the highest expression of VEGFA in αT3-1 cells, followed by miR-134 inhibitor + PBS group and blank vector control + SDF-1α group, the lowest is miR-134 mimics + PBS group (*P* < 0.01, **Figure 4A**). In addition, other proteins related to tumor proliferation/invasion were similar to VEGFA (*P* < 0.01, **Figures 4B–E**). As a consequence, SDF-1α inhibited the expression of the tumor suppressor gene miR-134 by promoting the expression of miR-134 target gene VEGFA to stimulate the proliferation and invasion ability of αT3-1 cells.

## Discussion

**Figure 5:**
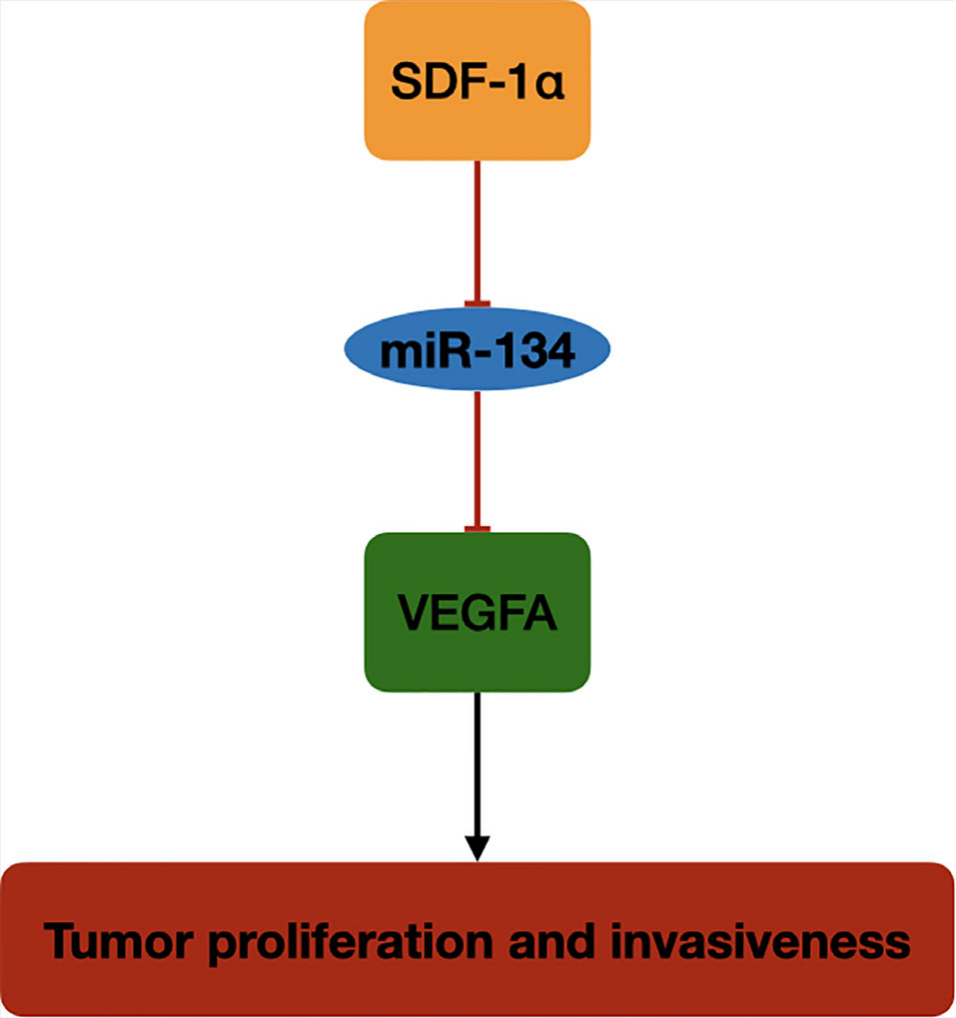
The schematic diagram of SDF-1α/miR-134/VEGFA axis in NF-PitNET. SDF-1α promotes VEGFA expression by inhibiting miR-134 expression in NF-PitNETs proliferation and invasiveness.

In this study, we investigated the role of miR-134 in NF-PitNET tumorigenesis and its interaction with tumor growth regulator, SDF-1α. The miR-134 expression was downregulated in mouse pituitary αT3-1 cells after SDF-1α treatment, with upregulation of its target gene VEGFA and proliferation and invasiveness. Also, miR-134 expression was found to be negatively correlated with proliferation and invasiveness in 29 human NF-PitNET tissues, with high expression level of SDF-1α and VEGFA in the invasive NF-PitNET. These findings indicate that miR-134 may function as a tumor suppressor in NF-PitNETs by suppressing the expression of VEGFA. Increased SDF-1α in NF-PitNETs inhibits miR-134 effect on VEGFA and plays an important role in tumor cell growth and invasiveness of NF-PitNETs (**Figure 5**).

MiR-134 is one of the microRNAs downstream of MEG3 in the DLK1-MEG3 gene cluster (14). Previous studies have shown that MEG3 is expressed in normal human pituitary gonadotrophs, but expression is lost in NF-PitNETs. Also, transfection and ectopic expression of MEG3 in human cancer cell lines can inhibit tumor cell proliferation (24). Further genetic analysis indicated that MEG3 is located in the DLK1-MEG3 gene cluster in human chromosome 14q32, and at least 80 imprinted genes have been found on this gene cluster so far. It contains three paternally expressed genes (PEGs), which are not normally expressed, and the rest belong to the MEGs, including long-chain non-coding RNAs such as MEG3, MEG8 and several microRNAs (25). The entire DLK1-MEG3 gene cluster is regulated by the upper-regulator of MEG3, intergenic differentially methylated region (IG-DMR) (26). While it was found that the IG-DMR was in a high methylation status in human NF-PitNET tissues, which suggests that it may be the main reason for the low specific expression of MEG3 in human NF-PitNET (27). The microRNAs in DLK1-MEG3 gene cluster located in the downstream of MEG3 were transcribed in the same direction as MEG3 (28). Subsequently, the following study proved the expression of six microRNAs (miR-134, miR-299-5p, miR-329, miR-370, miR-377-5p, and miR-432) was lost or reduced in NF-PitNETs, but normally expressed in normal pituitary and other types of pituitary tumors (14). Our previous study suggested miR-370–3p functions as a tumor suppressor gene by targeting HMGA2 in NF-PitNET and the tumor suppressor effect was regulated by SDF-1α (13). Also, our *in vitro* study demonstrated that SDF-1α exclusively silenced the expression of miR-134 and upregulated the target gene VEGFA in NF-PitNET.

Moreover, VEGFA plays an important role among the mechanisms that occur in pituitary tumorigenesis, angiogenesis for tumor growth, and was proposed as pituitary proangiogenic factor and possible therapeutic target (29). Tumor cells with upregulate VEGFA indicates high‐grade malignancy and poor outcome in different types of cancer. Patients with overexpression of VEGFA are prone to have larger tumor volumes, vascular hyperpermeability, and hemorrhage (30). We confirmed this by immunohistochemical staining (**Figure 1A**), the invasiveness was correlated with the VEGFA in the NF-PitNET. Meanwhile, we observed increased expression of Snail and Vimentin in cells treated with SDF-1 and miR-134 inhibitor in the western blot experiments. This result perhaps linking it up with the results from Transwell invasion assays considering that Snail and Vimentin overexpression are markers of the activation of epithelial-to-mesenchymal (EMT) (31), as well as the overexpression of MMPs, so the overexpression of these markers would suggest activation of EMT (a crucial pathway for cell migration and invasion) and therefore further support the increased invasiveness observed in Transwell invasion assay experiments (32, 33).

SDF-1α, also named chemokine (C-X-C motif) ligand 12 (CXCL12), is the single natural ligand of C-X-C chemokine receptor type (CXCR) 4 and CXCR7, plays an important roles in cancer cell proliferation, migration, and invasion (34). CXCL12 mRNA is expressed in about 2/3 of GH-secreting pituitary tumors and NF-PitNETs, with CXCR4 mRNA expressed in almost all these tumors (34). Further study proved that CXCL12/CXCR4 mediate the DNA synthesis and cell proliferation by human pituitary tumor primary cultures (35). And the expression of CXCR4 and CXCL12 were significantly higher in invasive PitNET compared with non-invasive PitNET specimens, evaluated by flow cytometry and immunohistochemical staining (36). Previous studies suggested SDF-1α contributes to vasculogenesis and mainly expresses in the areas with angiogenic activity (37). Several studies suggested that SDF-1α could upregulate the expression of VEGFA *via* SDF-1α/CXCR4 pathway (38, 39). Liang Z et al. (40) have reported that activation of PI3K/Akt pathway was also a critical part of CXCL12/CXCR4 signaling axis induced VEGFA expression increase. Interestingly, VEGFA can upregulate CXCL12 and CXCR4 mRNA expression, and contributes to U251 cell invasion (41). Kim et al. (42) use CXCR4 antagonists to inhibit GH secretion and also inhibit proliferation of GH-secreting pituitary tumor cells, which indicates targeting CXCL12 might be potentially also useful to NF-PitNETs. It was identified in our study by mouse cell lines that VEGFA expression and cell proliferation and invasiveness were significantly increased after SDF-1α treatment. In addition to the PI3K/Akt pathway, we used mmu-miR-134–5p inhibitor and mmu-miR-134–5p mimic proved the miR-134 was also an important part in SDF-1 α/VEGFA pathway.

## Limitations

Several limitations to our study deserve mention. Firstly, although our NF-PitNET samples were all negative for the detection of the GH, PRL, ACTH, FSH, LH by immunohistochemistry, we did not exclude the possibility that some of these tumors were not of gonadotroph lineage such as silent corticotroph, poorly differentiated PIT1-lineage, true null cell tumors, or other silent tumors of known cytodifferentiation. Secondly, SDF-1α/miR-134/VEGFA signaling pathway needs to be further clarified by luciferase assay. We have not performed *in vivo* experiments and other pituitary tumor cell line to further verify our result in NF-PitNET and we did not evaluate the effect of SDF-1 α on the methylation status of IG-DMR. Thirdly, the VEGFA expression level was only measured on protein level, thus, it remains uncover the molecular network among miR134 to mRNA of VEGFA. Future studies should include *in vivo* analyses with evaluation of methylation status of IG-DMR and mRNA alteration of SDF-1 α/miR-134/VEGFA signal pathway.

## Conclusion

The SDF-1α and VEGFA function important role in NF-PitNETs proliferation and invasiveness. The miR-134 expression was downregulated after SDF-1α treatment in mouse pituitary αT3-1 cells. Lower expression of miR-134 upregulated its target gene VEGFA expression and subsequently induced PitNET cell proliferation and invasiveness. The regulation of the SDF-1α/miR-134/VEGFA axis represents a novel mechanism in the pathogenesis of NF-PitNETs and may serve as a potential therapeutic target for the treatment of NF-PitNETs.

## Data Availability Statement

The datasets generated for this study are available on request to the corresponding authors.

## Ethics Statement

The studies involving human participants were reviewed and approved by the Ethics Committee of the Second Affiliated Hospital of Zhejiang University Medical College. The patients/participants provided their written informed consent to participate in this study.

## Author Contributions

XW and YF performed the experiments and analyzed the data. YF wrote the manuscript. XW edited the manuscript. YZ revised the manuscript. XG helped with the statistical analysis. KX and CL collected the samples. JZ and YH designed the study and revised the manuscript. All authors contributed to the article and approved the submitted version.

## Funding

This work was funded by the National Natural Science Foundation of China (No.81870964), Natural Science Foundation of Zhejiang Province (Nos. LY17H090012 and Q17H090013), Public Welfare Project of Zhejiang Provincial Department of Science and Technology (No. 2015C33192), and Rookie in the medical field of Zhejiang Province.

## Conflict of Interest

The authors declare that the research was conducted in the absence of any commercial or financial relationships that could be construed as a potential conflict of interest.
